# Non-Invasive Neuromodulation in the Rehabilitation of Pisa Syndrome in Parkinson's Disease: A Randomized Controlled Trial

**DOI:** 10.3389/fneur.2022.849820

**Published:** 2022-04-14

**Authors:** Roberto De Icco, Alessia Putortì, Marta Allena, Micol Avenali, Carlotta Dagna, Daniele Martinelli, Silvano Cristina, Valentina Grillo, Mauro Fresia, Vito Bitetto, Giuseppe Cosentino, Francesca Valentino, Enrico Alfonsi, Giorgio Sandrini, Antonio Pisani, Cristina Tassorelli

**Affiliations:** ^1^Movement Analysis Research Unit, IRCCS Mondino Foundation, Pavia, Italy; ^2^Department of Brain and Behavioral Sciences, University of Pavia, Pavia, Italy; ^3^Clinical Neurophysiology Unit, IRCCS Mondino Foundation, Pavia, Italy; ^4^Parkinson's Disease and Movement Disorders Unit, IRCCS Mondino Foundation, Pavia, Italy

**Keywords:** transcranial direct current stimulation (tDCS), neurorehabilitation, lateral trunk inclination, parkinsonism, movement analysis, movement disorders

## Abstract

**Background:**

Pisa syndrome (PS) is a frequent postural complication of Parkinson's disease (PD). PS poorly responds to anti-parkinsonian drugs and the improvement achieved with neurorehabilitation tends to fade in 6 months or less. Transcranial direct current stimulation (t-DCS) is a non-invasive neuromodulation technique that showed promising results in improving specific symptoms in different movement disorders.

**Objectives:**

This study aimed to evaluate the role of bi-hemispheric t-DCS as an add-on to a standardized hospital rehabilitation program in the management of PS in PD.

**Methods:**

This study included 28 patients with PD and PS (21 men, aged 72.9 ± 5.1 years) who underwent a 4-week intensive neurorehabilitation treatment and were randomized to receive: i) t-DCS (t-DCS group, *n* = 13) for 5 daily sessions (20 min−2 mA) with bi-hemispheric stimulation over the primary motor cortex (M1), or ii) sham stimulation (sham group, *n* = 15) with the same duration and cadence. At baseline (T0), end of rehabilitation (T1), and 6 months later (T2) patients were evaluated with both trunk kinematic analysis and clinical scales, including UPDRS-III, Functional Independence Measure (FIM), and Numerical Rating Scale for lumbar pain.

**Results:**

When compared to the sham group, the t-DCS group achieved a more pronounced improvement in several variables: overall posture (*p* = 0.014), lateral trunk inclination (*p* = 0.013) during upright standing position, total range of motion of the trunk (*p* = 0.012), FIM score (*p* = 0.048), and lumbar pain intensity (*p* = 0.017).

**Conclusions:**

Our data support the use of neuromodulation with t-DCS as an add-on to neurorehabilitation for the treatment of patients affected by PS in PD.

## Introduction

Pisa syndrome (PS) is a frequent postural complication of Parkinson's disease (PD), leading to higher motor and functional disability, gait impairment, higher occurrence of falls, lumbar pain, and cognitive impairment (1–4).

The main feature of PS is the lateral flexion of the trunk. This may be associated with a slight ipsilateral rotation of the trunk around the sagittal axis that leads to a higher and anterior position of the shoulder contralateral to the side of trunk inclination (4–6).

Pisa syndrome is a challenge for physicians as it poorly responds to antiparkinsonian drugs and only transiently improves with neurorehabilitation and botulinum toxin (2, 7, 8). Deep brain stimulation (DBS) may exert positive effects on PS, but its invasiveness and the lack of specifically designed clinical trials limit its application in clinical practice (9).

Several pathophysiological mechanisms have been hypothesized for the genesis of PS in PD, with central and peripheral mechanisms being involved (10). The central hypothesis suggests an important role in the imbalance in the dopaminergic outflow between left and right basal ganglia, leading to a postural trunk inclination toward the more denervated and less active striatum (1, 10). In this context, it is conceivable that an inter-hemispheric rebalance may translate into a clinical amelioration. Transcranial direct current stimulation (t-DCS) seems a proper tool for this purpose. t-DCS is a non-invasive electrical stimulation technique that modulates neural brain activity by means of low amplitude direct current trough surface electrodes (11). In PD, M1 stimulation may modulate basal ganglia outflow through at least two different mechanisms: i) an increase in M1 excitability may potentiate the underlying pallido-thalamocortical network, and ii) the dopamine release in the basal ganglia may increase after M1 anodal cortical stimulation through potentiation of the glutamatergic cortico-striatal pathway (11–13). Our working hypothesis is that a bi-hemispheric t-DCS approach may re-equilibrate the impaired dopaminergic outflow between left and right basal ganglia, thus resulting in a clinical improvement of the trunk inclination.

It is worth noting that the central hypothesis is supported by animal and human data. Stimulation of the striatum in animal models (cholinergic or dopamine agonist, electric stimulation, or subthalamic nucleus ablation) induced a trunk inclination contralateral to the stimulated striatum (14, 15). By contrast, experimentally induced unilateral nigrostriatal denervation induced an ipsilateral trunk inclination (16, 17).

These pre-clinical observations are consistent with clinical evidence. PS is more prevalent in PD patients with high motor asymmetry, and in the majority of patients, the side of PS inclination is ipsilateral to the less affected side at PD onset (2, 3, 6). Unilateral surgical therapeutic strategies (such as pallidotomy and subthalamic nucleus ablation) were complicated by the onset of a PS contralateral to the surgical site (18). Most important, DBS, which clearly acts at the central level, proved effective in several case series (9, 19, 20).

Transcranial direct current stimulation delivered over the primary motor cortex (M1) has been tested in several movement disorders with interesting, although sometimes conflicting, results (21). In idiopathic PD, t-DCS improved freezing of gait (FOG) and balance (22, 23), upper limbs bradykinesia, writing and sequential movements (24, 25), and severity of dyskinesia (26). However, this technique has never been tested in the management of patients with PD along with PS, although the central pathogenetic hypothesis appears to be particularly suitable to this approach.

In this randomized, sham-controlled study, we aimed to evaluate the efficacy of a bi-hemispheric t-DCS as an add-on to a standardized in-hospital rehabilitation program in the management of PS in PD. The primary outcome of the study was the improvement of overall trunk posture in the upright standing position at the end of the rehabilitation.

## Materials and Methods

### Subjects

This study involved 28 patients affected by PD and PS who were consecutively enrolled among those attending the Neurorehabilitation Department of the Istituto di Ricovero e Cura a Carattere Scientifico (IRCCS) Mondino Foundation (Pavia, Italy) between January 2018 and August 2020. No patients withdrew from the study.

Idiopathic PD was diagnosed according to the Movement Disorders Society's clinical diagnostic criteria for PD (27). PS was clinically diagnosed according to the following criteria (1, 3, 28): lateral inclination of the trunk of at least 10°, ipsilateral axial rotation of the trunk, worsening of the postural disorder during standing position, and gait with an almost complete resolution of trunk inclination by passive mobilization or supine positioning. Inclusion criteria were as follows: age between 18 and 80 years; Hoehn & Yahr stage between II and III; Mini-Mental State Examination score above 24. The exclusion criteria were as follows: history of major psychiatric or other neurological conditions, spine surgery, other neurological, rheumatological, or orthopedic spine diseases, ongoing or previous treatment with neuroleptic drugs, botulinum toxin treatment in the previous year, and any change in dose or regimen of the anti-parkinsonian therapy in the last month before enrolment. All patients enrolled were naïve to hospital intensive neurorehabilitation, as well as to neuromodulation.

### Study Procedures

The study was a randomized, double-blind, controlled trial that aimed to assess the efficacy of five daily sessions of bi-hemispheric t-DCS in add-on to an in-hospital rehabilitation protocol in patients affected by PS and PD.

At hospital admission (T0), the patients underwent a complete neurological examination, a baseline kinematic analysis of trunk movement, and the administration of a set of clinical scales.

Patients were then randomly assigned to “t-DCS” or “sham” treatment according to a block randomization method (6 blocks; 6 patients per block) and referred to as the t-DCS group and the sham group, respectively.

The randomization procedure was performed by the local Biodata Center, whose staff was not involved in the study procedures. The Biodata Center staff communicated the randomization code directly to the technician responsible for the t-DCS-related procedures, who was not involved in the subsequent part of the protocol.

A unique randomization list was generated before the start of the enrolment. The randomization code remained in the hospital during the study and was not available to the investigators until the study was completed and data were analyzed. The randomization code was stored in a light-sealed envelope and might be broken in case of need for patient safety. There was an envelope for each patient so that the overall randomization code was not revealed in case an envelope was broken.

We used a bi-hemispheric t-DCS approach according to previously published protocols (29, 30).

Parallel to neuromodulation, all patients were treated with a standardized 4-week rehabilitation program with 6 sessions per week lasting 90 min each (Supplementary Material 1).

**Figure 1:**
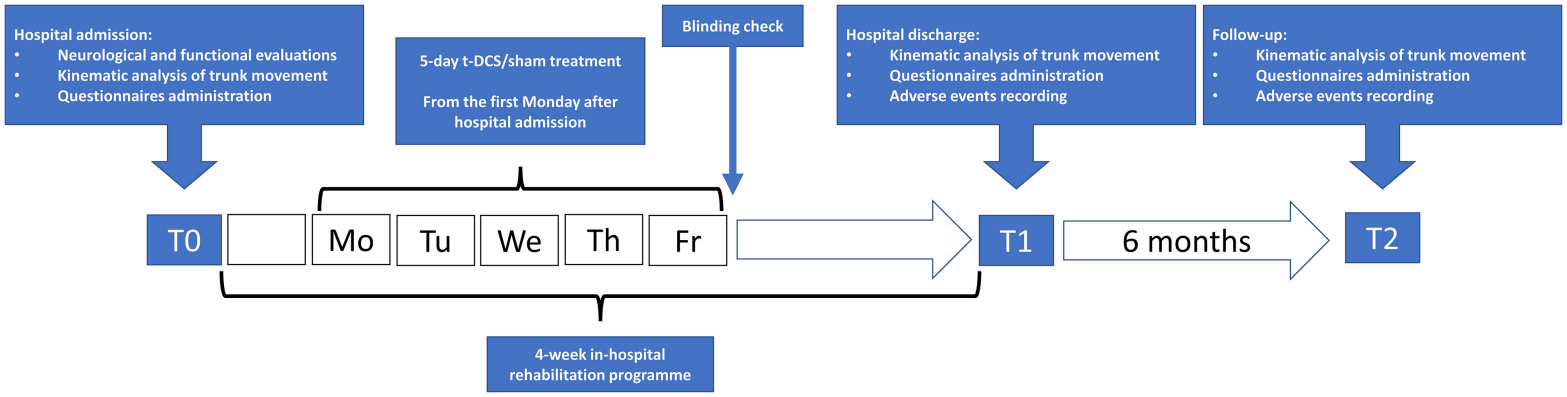
Flowchart of study procedures. t-DCS: patients randomized to transcranial direct current stimulation (*n* = 13). sham: patients randomized to sham stimulation (*n* = 15).

The kinematic analysis of trunk movement and the administration of the set of clinical scales were repeated at the end of the rehabilitation program (T1) and 6 months later (T2) (Figure 1).

All patients received their individualized anti-parkinsonian drug therapy, whose dosing scheme was kept stable during the whole study period.

In all patients, PS was not responsive to the anti-parkinsonian drug therapy and its severity did not fluctuate during the day, as we clinically confirmed during the 4-week hospital observation period.

The local ethics committee approved the study (p-20190052462), and all participants signed a written informed consent before enrolment. The trial was registered at www.clinicaltrials.gov (NCT04620863). The authors agree to share anonymized data from this analysis upon reasonable request from qualified investigators. The study was completed in September 2020.

### Technical Aspects of Transcranial Direct Current Stimulation (t-DCS)

Neuromodulation was delivered *via* a specific battery-driven direct current stimulator (Newronika HDCstim, Newronika s.r.l., Milan, Italy) by an expert technician (VG) who was not otherwise involved in the management of the patients. The current was transferred by a saline-soaked pair of surface sponge electrodes (3 × 3 cm for both anode and cathode). t-DCS was always administered in the morning between 8:00 a.m. and 12:00 p.m. The stimulation was delivered within 3 hours after the first L-dopa administration of the day during the ON phase.

All the participants received daily sessions for 5 consecutive days (20 min per session with a 2 mA intensity). The stimulation started from the first Monday after hospital admission, indeed it was always started in the first seven days of rehabilitation. The primary motor cortex (M1) was identified using the International 10–20 system for C3 (left M1) or C4 (right M1).

We used a bi-hemispheric t-DCS approach according to previously published protocols (29, 30):

- cathodal stimulation over M1 contralateral to the side of trunk inclination (inhibition of the more active hemisphere/striatum);
- anodal stimulation over M1 ipsilateral to the side of PS (potentiation of the more denervated hemisphere/striatum).

In the sham group, patients underwent the same number of sessions that lasted 20 minutes, but the stimulation intensity was set according to a ramping up/ramping down method and delivered only in the first and last 30 seconds of each session (31, 32). This stimulation paradigm was insufficient to produce a meaningful therapeutic effect, but it mimicked the possible initial tingling sensation associated with active stimulation. These procedures adequately blinded participants to their group allocation (32, 33). At the end of the 5-day stimulation period, a blind check was performed. During the hospital observation period, as well as at T2, patients were monitored for the appearance of any side effects.

### Kinematic Analysis of Trunk Movement

**Figure 2:**
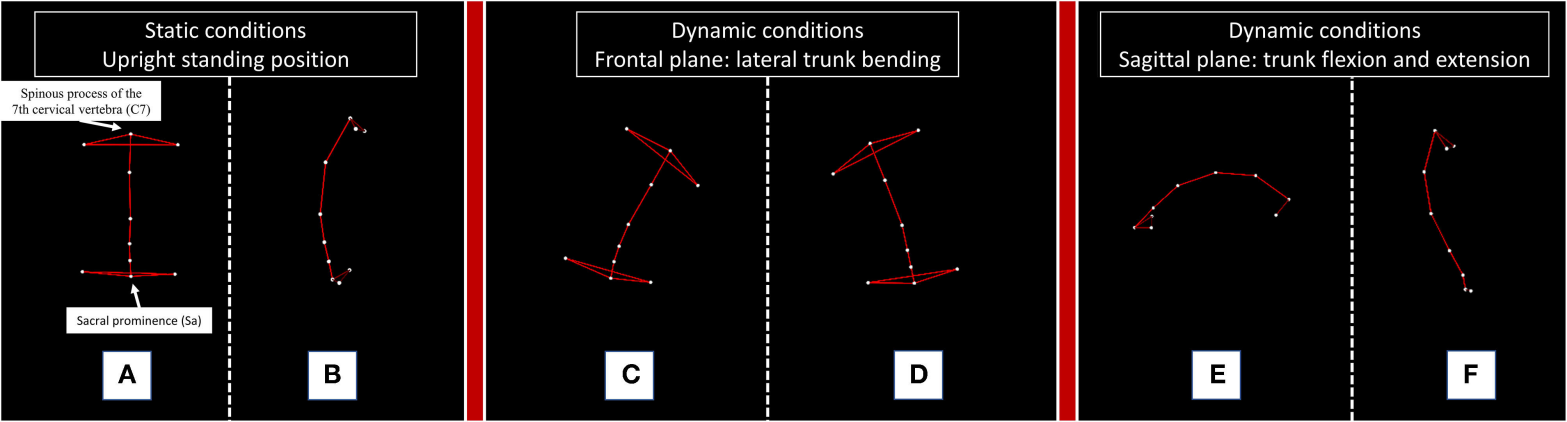
Kinematic analysis of trunk movement in static and dynamic conditions performed by a representative subject. The figure illustrates the static and dynamic tasks performed by a representative healthy subject. C7: the spinous process of the 7th cervical vertebra; Sa: sacral prominence. For the measurement of lateral **(A)** and anterior **(B)** trunk deviation, we considered the absolute deviation of the “C7-Sa” segment from the vector perpendicular to the floor of our movement analysis laboratory during the static upright standing position. For the dynamic tasks, we calculated the range of motion (ROM) of the trunk, defined as the maximum angle described by the C7-Sa segment starting from the upright standing position to the end of a right **(C)** or left **(D)** trunk bending, a forward trunk flexion **(E)** and a posterior trunk extension **(F)**.

Kinematic analysis of trunk was performed with a 4-camera optoelectronic system (SMART DX 400, BTS Engineering, Milan, Italy) (2, 7). All patients were evaluated in the afternoon between 3:00 p.m. and 6:00 p.m. and always during a clinically confirmed ON phase. We studied the patients in static and dynamic conditions. The upright standing position was recorded with subjects standing with their feet 10 cm apart and their arms lying along their trunk. For the dynamic tasks, patients were asked to perform lateral trunk bending on both sides, forward trunk flexion and a posterior trunk extension (Figure 2).

The absolute deviation and the range of motion (ROM) of the C7-Sacrum segment (C7-Sa) were calculated according to a previously validated two-landmark system (Supplementary Material 2) (2, 7).

The following parameters were recorded: lateral trunk inclination in the upright standing position (Stat Bend); anterior trunk flexion in the upright standing position (Stat Flex); total postural alteration in the upright standing position (Stat Tot: Stat Bend + Stat Flex); ROM of trunk bending ipsilateral to the side of trunk deviation (ROM Ips); ROM of trunk bending contralateral to the side of trunk deviation (ROM Con); ROM of anterior trunk flexion (ROM Flex); ROM of posterior trunk extension (ROM Ext); and total ROM of the trunk (ROM Tot: ROM Ips + ROM Con + ROM Flex + ROM Ext).

### Clinical Scales for Motor Disability, Functional Independence, and Lumbar Pain

Parkinson's disease-related motor disability was assessed through the Unified Parkinson's Disease Rating Scale—Part III (UPDRS-III) (34). The item 3.13 Posture of the UPDRS-III scale was used as an overall measure of the postural alteration of PS in PD. Functional independence was measured through the Functional Independence Measure (FIM) (35), while lumbar pain severity was rated according to a 0 to 10 numerical rating scale (NRS).

### Statistical Analysis

The sample size was calculated with the online platform www.openepi.com and based on the difference between the t-DCS and sham groups in the Stat TOT at T1 (we considered as clinically meaningful a between-groups difference of at least 15 ± 10%). The computation was made with the following parameters: CI (two-sided): 95%; power: 80%; ratio of sample-size: 1:1; mean difference: 15; and *SD*: 10. The minimum sample size suggested was 24 patients (12 patients per group).

The SPSS version 21 (Windows, IBM, N.Y., USA) was used for all the computations. The Kolmogorov-Smirnov test confirmed a normal distribution of our data. Continuous variables were presented as “mean ± *SD*,” while categorical data were presented as “number (in percentage).” For the statistical analysis, the data were normalized to a 100% baseline and expressed at T1 and T2 as percentage modifications.

Between groups, a comparison was performed with a Student's *t*-test for independent samples. Statistical association among categorical variables was tested with Pearson χ^2^ test or Fisher exact test, if appropriate.

The main analysis was performed with mixed-model ANOVA with two fixed factors: TIME (as in time) (within-subjects factor with 3 levels: T0 vs. T1 vs. T2) and STIM (as in stimulation) (between-subjects factor with 2 levels: t-DCS group vs. sham group), followed by a *post hoc* Bonferroni's correction for intra-group comparisons. If a significant interaction TIMExSTIM was found, a *post hoc* analysis was separately performed for the t-DCS group and the sham group.

The primary endpoint was the difference between groups in the percentage modification of Stat Tot at T1 and T2. Stat Tot was selected as the primary outcome measure because it may reflect more comprehensively the full range of improvement of trunk alignment associated with our experimental rehabilitative intervention. Secondary endpoints were the following: Stat Bend; ROM Tot; UPDRS-III; item 3.13 Posture of the UPDRS-III scale; FIM; NRS.

All other endpoints (Stat Flex; ROM Ips; ROM Con; ROM Flex; ROM Ext) were considered exploratory. To rule out a significant association between the clinical and demographic features and the primary outcome, we performed an exploratory ANOVA for repeated measures test with factor TIME and factors such as gender, type of PD at onset, the most affected side of PD at onset, and the side of trunk inclination. Exploratory analyses are provided in Supplementary Material 3.

The level of significance was set at α = 0.05 and always corrected for multiple comparisons where appropriate.

## Results

### Clinical and Demographic Features of the Study Population

Transcranial direct current stimulation group (*n* = 13, 9 men, 71.9 ± 5.2 years old) and sham group (*n* = 15, 12 men, 73.7 ± 5 years old) were comparable for clinical and demographic features (Table 1).

**Table 1:** Clinical and demographic features, kinematic analysis of trunk movement, and scores of clinical scales of study populations at baseline.

		**All patients**	**t-DCS group**	**Sham group**	***p***
*N*		28	13	15	
**Clinical and demographic features**					
Age (years)		72.9 ± 5.1	71.9 ± 5.2	73.7 ± 5.0	0.377
Sex	Male	21 (75.0%)	9 (69.2%)	12 (80.0%)	0.512
	Female	7 (25.0%)	4 (30.8%)	1 (20.0%)	
PD duration (years)		9.3 ± 7.4	8.7 ± 5.8	9.8 ± 8.8	0.702
Most affected side at PD onset	Left	8 (28.6%)	4 (30.8%)	4 (26.7%)	0.706
	Right	13 (46.4%)	5 (38.5%)	8 (53.3%)	
	Symmetric	7 (25.0%)	4 (30.8%)	3 (20.0%)	
Type of PD at onset	Tremor-dominant	7 (25.0%)	4 (30.8%)	3 (20.0%)	0.587
	Akinetic-rigid	17 (60.7%)	8 (61.5%)	9 (60.0%)	
	Complete	4 (14.3%)	1 (7.7%)	3 (20.0%)	
Ongoing anti-parkinsonian therapy	Levodopa	28 (100%)	12 (100%)	13 (100%)	-
	Dopamine agonist	22 (78.6%)	10 (76.9%)	12 (80.0%)	0.843
	COMT inhibition	16 (57.1%)	7 (53.8%)	9 (60.0%)	0.743
	MAO-B inhibition	9 (67.9%)	4 (30.8%)	5 (33.3%)	0.885
Levodopa equivalent daily dose (mg)		980.6 ± 280.7	963.9 ± 236.7	999.7 ± 333.4	0.743
Time elapsed since first report of lateral trunk inclination (years)		3.0 ± 1.9	2.8 ± 2.2	3.1 ± 1.7	0.770
Side of trunk inclination	Left	13 (46.4%)	7 (53.8%)	6 (40.0%)	0.705
	Right	15 (53.6%)	6 (46.2%)	9 (60.0%)	
**Kinematic analysis of trunk movement (degree)**					
Stat Tot		41.9 ± 18.7	42.3 ± 16.6	41.5 ± 20.9	0.920
Stat Bend		15.9 ± 7.2	16.4 ± 4.4	15.4 ± 9.1	0.732
ROM Tot		111.3 ± 31.1	102.7 ± 32.4	118.7 ± 29.1	0.179
**Score of clinical scales**					
UPDRS-III		30.6 ± 8.8	29.5 ± 10.1	31.7 ± 7.7	0.520
Item 3.13 Posture of the UPDRS-III	1	2 (7.1%)	1 (7.7%)	1 (6.7%)	0.892
	2	11 (39.3%)	5 (38.5%)	6 (40.0%)	
	3	12 (42.9%)	5 (38.5%)	7 (46.7%)	
	4	3 (10.7%)	2 (15.4%)	1 (6.7%)	
FIM		93.8 ± 15.9	87.8 ± 15.3	99.0 ± 14.9	0.061
Patients with lumbar pain		15 (53.6%)	7 (53.8%)	8 (53.3%)	0.638
NRS		3.3 ± 3.2	3.5 ± 3.4	3.1 ± 3.3	0.758

*PD, Parkinson's disease; t-DCS, patients randomized to transcranial direct current stimulation (n = 13). sham: patients randomized to sham stimulation (n = 15). Clinical and demographic features: Time elapsed since the first report of lateral trunk inclination: time in years elapsed since the occurrence of a more or less marked lateral trunk inclination noted by the patients or reported in the physician's notes. COMT, catechol-O-methyltransferase; MAO-B, monoamine oxidase B. Kinematic analysis of movement: Stat Tot: Total postural alteration in the upright standing position. Stat Bend: Lateral trunk inclination in the upright standing position. ROM: range of motion. ROM Tot: global range of motion of the trunk. Clinical scales: UPDRS-III: Unified Parkinson's Disease Rating Scale—Part III—Motor examination. FIM, Functional Independence Measure. NRS: 0–10 numerical rating scale for lumbar pain severity*.

In the subset of patients with unilateral or asymmetric motor symptoms at PD onset (21 subjects, 9 in the t-DCS group, and 12 in the sham group), the lateral trunk inclination was toward the less affected side in 12 (57.1%) patients, of which 4 (44.4%) were in the t-DCS group and 8 (66.7%) were in the sham group (*p* = 0.396).

### Kinematic Analysis of Movement at Baseline

The postural alterations in an upright standing position and the ROM tot were comparable between groups at baseline: *Stat Tot*: *p* =0.92; *Stat Bend*: *p* =0.732; *ROM Tot*: *p* =0.179 (Table 1).

### Effects of t-DCS and Sham Treatments on the Kinematic Analysis of Trunk Parameters in an Upright Standing Position

**Figure 3:**
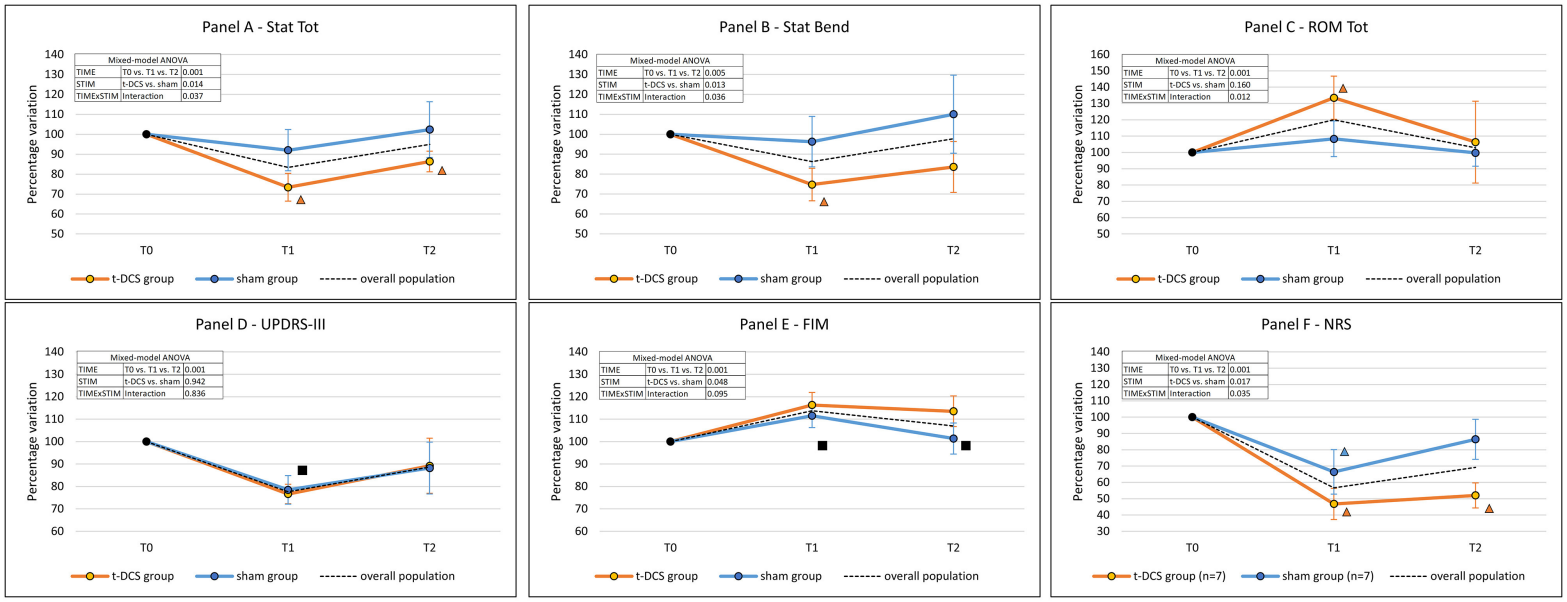
Effects of t-DCS and sham treatments on the kinematic analysis of trunk parameters and clinical scales scores. t-DCS: patients randomized to transcranial direct current stimulation (*n* = 13). sham: patients randomized to sham stimulation (*n* = 15). ROM: range of motion. Error bars: standard deviation. **(A)** Stat Tot: total postural alteration in the upright standing position. **(B)** Stat Bend: lateral trunk inclination in the upright standing position. **(C)** ROM Tot: sum of trunk ROMs of the four dynamic tasks. **(D)** UPDRS-III: Unified Parkinson's Disease Rating Scale—Part III—Motor examination. **(E)** FIM, Functional Independence Measure. **(F)** NRS: 0–10 numerical rating scale NRS for lumbar pain severity. Mixed-model ANOVA: factor “TIME” is the expression of the efficacy of the rehabilitative treatment in the overall population; factor “STIM” is the expression of the comparison between t-DCS and sham groups across all time-points. If the “TIMExSTIM” interaction was not significant, a *post-hoc* analysis was performed in the overall population: ■ = time-point vs. T0: *p* < 0.05; in case of a significant “TIMExSTIM” interaction, a *post-hoc* analysis was separately performed for the t-DCS group and the sham group: Δ = time-point vs. T0: *p* < 0.05 (the color identifies the group).

The total postural alteration in the upright standing position (*Stat Tot* – primary outcome) improved after rehabilitation in the overall study population (TIME: *p* = 0.001), being more pronounced in the t-DCS group (STIM: *p* = 0.014). At *post hoc* intra-group analyses (TIMExSTIM: *p* = 0.037), the *Stat Tot* parameter was not modified in the sham group (*p* = 0.14). By contrast, in the t-DCS group, *Stat Tot* was reduced both at T1 (*p* = 0.001 vs. T0) and at T2 (*p* = 0.001 vs. T0) when compared to baseline (Table 2 and Figure 3A).

**Table 2:** Effects of t-DCS and sham treatments on kinematic analysis of trunk parameters and clinical scales.

	**T0**	**T1**			**T2**			**Mixed-model ANOVA**		
		**All patients**	**t-DCS**	**sham**	**All patients**	**t-DCS**	**sham**	**TIME**	**STIM**	**TIMExSTIM**
***N***	**28**	**28**	**13**	**15**	**28**	**13**	**15**			
**Static upright standing position and range of motion of active dynamic tasks**										
Stat Tot (%)	100	83.4 ± 9.9	73.4 ± 6.9	92.0 ± 10.3	94.9 ± 11.4	86.4 ± 5.2	102.4 ± 13.9	0.001	0.014	0.037
Stat Bend (%)	100	86.3 ± 11.9	74.8 ± 8.2	96.3 ± 12.7	97.8 ± 17.8	83.6 ± 12.8	110.0 ± 19.6	0.005	0.013	0.036
ROM Tot (%)	100	120.0 ± 13.4	133.5 ± 13.2	108.3 ± 10.9	102.8 ± 17.8	106.3 ± 25.1	99.7 ± 8.2	0.001	0.160	0.012
**Score of clinical scales**										
UPDRS-III	100	77.6 ± 5.4	76.6 ± 4.3	78.5 ± 6.4	88.7 ± 11.7	89.2 ± 12.3	88.2 ± 11.5	0.001	0.942	0.836
FIM	100	113.8 ± 5.5	116.3 ± 5.6	111.5 ± 5.3	107.0 ± 7.4	113.5 ± 6.8	101.4 ± 6.9	0.001	0.048	0.095
NRS	100	56.6 ± 12.4	46.8 ± 9.6	66.4 ± 13.6	69.2 ± 13.3	52.0 ± 7.7	86.4 ± 12.3	0.001	0.017	0.035

*t-DCS, patients randomized to transcranial direct current stimulation (n=13); sham, patients randomized to sham stimulation (n = 15); Stat Tot, Total postural alteration in the upright standing position; Stat Bend, Lateral trunk inclination in the upright standing position; ROM, range of motion; ROM Tot, global range of motion of the trunk; Clinical scales: UPDRS-III, Unified Parkinson's Disease Rating Scale—Part III—Motor examination; FIM, Functional Independence Measure; NRS: 0–10 numerical rating scale for lumbar pain severity. Mixed-model ANOVA: factor “TIME” is the expression of the efficacy of the rehabilitative treatment in the overall population; factor “STIM” is the expression of the comparison between t-DCS and sham groups across all time-points; a significant TIMExSTIM interaction is the expression of a difference between t-DCS and sham groups as well as the difference in the persistence of the effects between t-DCS and sham groups over time*.

*Stat Bend* improved after the rehabilitation program in the overall study population (TIME: *p* = 0.005). The reduction of *Stat Bend* was more pronounced in the t-DCS group (STIM: *p* = 0.013). The *post hoc* intra-group analysis (TIMExSTIM: *p* = 0.036) showed that the *Stat Bend* parameter was not modified in the sham group (*p* = 0.375); by contrast, in the t-DCS group the lateral trunk inclination was reduced at T1 (*p* = 0.001 vs. T0), but the improvement was not retained at T2 (*p* = 0.118 vs. T0) (Table 2 and Figure 3B).

The lateral trunk inclination fell below the 10° diagnostic cut-off in 5 (38.5%) patients of the t-DCS group and in 3 (20%) patients of the sham group at T1 (*p* = 0.41), and in 4 (30.8%) patients of the t-DCS group and in 1 (6.7%) patient in the sham group at T2 (*p* = 0.153).

### Effects of t-DCS and Sham Treatments on the Kinematic Analysis of Trunk Parameters During Dynamic Tasks

The *ROM Tot* significantly increased in the overall study population (TIME: *p* = 0.001). A different behavior over time was described between groups (TIMExSTIM: *p* = 0.012, STIM: *p* = 0.160). Specifically, in the t-DCS group, we found an improvement at T1 (*p* = 0.003 vs. T0), which was not retained at T2 (*p* = 1.000 vs. T0), while no improvement was detected in the sham group at either time point (Figure 3C and Table 2).

### Clinical Scales at Baseline

The two study groups showed similar levels of functional independence (FIM: *p* = 0.061), motor disability (UPDRS-III: *p* = 0.52), and postural alteration as measured by posture-specific item 3.13 of the UPDRS-III scale (*p* = 0.79) (Table 2).

Lumbar pain was reported in 7 (53.8%) patients in the t-DCS group and in 8 (53.3%) in the sham group (*p* = 0.638). Pain severity was comparable between groups (NRS: *p* =0.758) (Table 2).

### Effects of t-DCS and Sham Treatments on Motor Disability, Functional Independence, and Lumbar Pain

Unified Parkinson's Disease Rating Scale—Part III score was reduced at T1 (*p* = 0.001 vs. T0) but not at T2 (*p* = 0.056 vs. T0) in the overall population (TIME: *p* = 0.001), without differences between the t-DCS and sham groups (STIM: *p* = 0.942, and TIMExSTIM: *p* = 0.836) (Figure 3D and Table 2).

The posture-specific item 3.13 of the UPDRS-III scale showed a better score distribution in the t-DCS group when compared to the sham group at T1 (*p* = 0.019), but not at T2 (*p* = 0.355) (Supplementary Table 3).

Functional Independence Measure score improved after rehabilitation in both groups (TIME: *p* = 0.001) at T1 and also at T2, with a greater increase in the t-DCS group (STIM: *p* = 0.048) (Figure 3E and Table 2).

The overall percentage of patients with lumbar pain did not change throughout the study (*p* = 0.105). In the subgroup of patients reporting lumbar pain at all time-points (7 in both groups), the improvement in pain scores was more pronounced in the t-DCS group when compared to the sham group (STIM: *p* = 0.017). Remarkably, the persistence in NRS reduction was different between groups (TIMExSTIM: *p* = 0.035) and still significantly lower at T2 (*p* = 0.001 vs. T0) only in the t-DCS group (Figure 3F and Table 2).

### Blinding Check and Adverse Events

Finally, 7 out of 13 patients (53.8%) in the t-DCS group and 8 out of 15 patients (53.3%) in the sham group believed that they received active treatment (*p* = 0.978). All patients tolerated well the stimulation and no side effects were reported.

## Discussion

In this randomized, sham-controlled study, we evaluated the effects of a bi-hemispheric t-DCS approach as an add-on to standardized 4-week hospital neurorehabilitation in the management of PS in PD.

The overall posture improved with neurorehabilitation, more markedly and, for some outcome measures, also more persistently over time in the t-DCS group. The reduction in lateral trunk inclination (the core feature of PS) was indeed more pronounced in the t-DCS group at the end of the rehabilitation program. Similarly, the increase in the global ROM of the trunk was more pronounced in the t-DCS group at T1. The postural and mobility trunk improvement was coupled with a reduction of motor disability at T1 in the overall population, and an increase in FIM score at both T1 and T2 was more pronounced in the t-DCS group. Although the percentage of patients reporting lumbar pain was not reduced, pain severity was lower and the benefit persisted longer in the t-DCS group.

The efficacy of bi-hemispheric t-DCS has been previously reported in focal dystonia (30, 36, 37), while, to the best of our knowledge, this neuromodulation approach has never been tested in PS in PD. Indeed, the hypothesized role of basal ganglia output asymmetry in PS appears to be a reasonable substrate for the proposed bi-hemispheric approach (10), and therefore we adopted the bi-hemispheric t-DCS approach to re-equilibrate the imbalance between left and right basal ganglia, which represents the neuronal substrate of the central pathophysiological hypothesis of PS in PD (10).

The following mechanisms were hypothesized the explain the circuitries involved in basal ganglia modulation after M1 neuromodulation: i) an increase/decrease in M1 excitability may modulate the pallido-thalamocortical pathway, and ii) the dopamine release in the basal ganglia after M1 anodal cortical stimulation may potentiate/inhibit the glutamatergic cortico-striatal pathway (11–13). In this frame, the more pronounced improvement observed in the t-DCS group at T1, and its partial retention at T2, can be ascribed to enhanced neuronal plasticity induced by cortical stimulation, which may lead to long-lasting after-effects such as long-term potentiation or long-term depression (38, 39).

As previously mentioned, several case series suggest how DBS may exert positive effects on PS, but its invasiveness and the lack of specifically designed and well-powered clinical trials have limited its application in clinical practice (9, 19, 20).

Only six studies were specifically designed to test the efficacy of non-invasive therapies (neurorehabilitation, botulinum toxin, or lidocaine injections) in patients with PS and PD (2, 7, 8, 40–42), and among these only two were randomized controlled trials (2, 40). Three studies used a kinematic analysis of movement for recording the outcome measures (2, 7, 41), but only two of them evaluated trunk ROM during dynamic tasks (2, 7). The improvement achieved in our t-DCS group is consistent with previous data reporting a reduction between 30 and 50% of lateral trunk inclination and a comparable increase of ROM of trunk bending at the end of a rehabilitation program (2, 7, 8, 40–42). The new and inspiring aspect of our present findings is that the addition of t-DCS to neurorehabilitation induced a more persistent improvement in trunk posture. This result has a great clinical significance because the persistence of the improvement is one of the critical issues in the management of PS in PD. In this frame, it is tempting to hypothesize that the observed benefit may possibly be further extended over time when considering the possibility of repeating t-DCS stimulation in the ambulatory setting in association with a tailored physical exercise program at home.

Some limitations must be acknowledged for a comprehensive interpretation of our results. First, the small sample size of the present study does not allow us to infer definitive conclusions. In addition, based on our inclusion/exclusion criteria, we enrolled a population without cognitive impairment and without the compelling need for frequent adjustments of the anti-parkinsonian treatment, which further reduced our cohort. Yet, the adaptability of t-DCS is one of its intrinsic limits, wherein although we adopted a bi-hemispheric approach, we cannot exclude that other stimulation paradigms might induce comparable or even better results. For instance, a longer t-DCS stimulation during the hospital setting may induce more pronounced or more persistent effects. Nonetheless, a different study paradigm, namely t-DCS alone or delivered immediately before or after the rehabilitative period, may exert different pathophysiological effects.

Despite these limitations, this study warrants further trials on larger cohorts, in order to confirm our findings.

In conclusion, t-DCS potentiates the effects of neurorehabilitation in the management of PS in PD. Our data support the use of a bi-hemispheric t-DCS approach as an add-on to neurorehabilitation in the management of patients affected by PS and PD. t-DCS may represent a therapeutic alternative, being a non-invasive, well-tolerated, easily repeatable, and low-cost technique.

## Data Availability Statement

The raw data supporting the conclusions of this article will be made available by the authors, without undue reservation.

## Ethics Statement

The studies involving human participants were reviewed and approved by Local Ethics Committee of Pavia (p-20190052462). The patients/participants provided their written informed consent to participate in this study.

## Author Contributions

RD: study concept or design, analysis or interpretation of data, writing of the first draft, and RDI takes responsibility for the integrity of the data and the accuracy of the data analysis. APu: acquisition of data, analysis or interpretation of data, drafting, and revision of the manuscript for content. MAl, MAv, GC, and FV: patients enrolment, drafting, and revision of the manuscript for content. CD and SC: patients enrolment and acquisition of data. DM: patients enrolment, acquisition of data, drafting, and revision of the manuscript for content. VG: responsible for t-DCS related procedures. MF: acquisition and analysis of kinematic movement analysis data. VB: acquisition and analysis of data, drafting, and revision of the manuscript for content. EA, GS, and APi: critical analysis of findings and review of the manuscript. CT: study concept or design, drafting, and revision of the manuscript for content. All authors contributed to the article and approved the submitted version.

## Funding

This study was funded by the Italian Ministry of Health (Ricerca Corrente 2018–2020).

## Conflict of Interest

RD, APu, MAl, MAv, CD, DM, SC, VG, MF, VB, GC, FV, GS, and EA report no funding in the preceding 12 months. APi holds grants that are not related to the subject of the present study, and he reports no biomedical financial interests or potential conflicts of interest. CT received honoraria for their participation in advisory boards or for oral presentations from Allergan, ElectroCore, Eli-Lilly, Novartis, and Teva. CT has no ownership interest and does not own stocks of any pharmaceutical company. CT serves as Chief Section Editor of Frontiers in Neurology—Section Headache Medicine and Facial Pain and on the editorial board of The Journal of Headache and Pain.

## Publisher's Note

All claims expressed in this article are solely those of the authors and do not necessarily represent those of their affiliated organizations, or those of the publisher, the editors and the reviewers. Any product that may be evaluated in this article, or claim that may be made by its manufacturer, is not guaranteed or endorsed by the publisher.
