# Supplementary material for: Non-Invasive Neuromodulation in the Rehabilitation of Pisa Syndrome in Parkinson's Disease: A Randomized Controlled Trial
Source: Front Neurol. 2022 Apr 14;13:849820. doi: 10.3389/fneur.2022.849820 (PMC9046718; doi:10.3389/fneur.2022.849820)
Supplement: Supplementary file 1 [file Table_1.docx]

**Supplementary Material 1 - In-hospital rehabilitation program**

All patients enrolled were treated with a specific and standardized in-hospital rehabilitation program which was focused on the rehabilitation of the trunk postural disorder [7,8]. According to the present national regional law (n° X/ 1980, 20th June 2014), all patients were treated in the ON condition with 90-minute daily sessions, 6 days a week (Monday to Saturday) for four weeks. The rehabilitative treatment was delivered in the morning or in the afternoon according to the patients’ needs and preferences, and to the organization of the clinical activities of the Neurorehabilitation Department.

Each session was structured as follow:

- 10 minutes of cardiovascular warm-up activities: intersegmental coordination exercises; exercises to release shoulder and pelvic girdle; pelvic anteversion and retroversion movements to improve diaphragmatic respiration (supine position); breathing exercises to promote expansion of the chest;

- 15 minutes of stretching exercises: exercises to stretch the muscles of the posterior kinematic chain; exercises to stretch the pectoralis muscles; exercises to stretch the ischio-cruralis muscles; assumption of the prone position, sitting on heels and stretching the arms out in front; the “bridge” exercise to stretch the muscles of the anterior abdominal wall, glutei, quadriceps and hamstring; exercises to stretch lumbar muscles (in the supine position, each knee, in turn, is brought to the chest);

- 15 minutes of strengthening exercises in a functional context: exercises to strengthen the dorsal muscles (arms extended and hands outstretched as though to take something); lateral bending (arms lying along the body and hands reaching down as though to pick up something); stretching using the wall bars;

- 20 minutes of gait training: overground gait training (forwards, backwards, and lateral); walking on the spot;

- 15 minutes of balance training: path with obstacles; balance exercises performed in order of difficulty (heel-to-toe walking, lateral walking crossing the legs, walking along a path on surfaces of different texture);

- 15 minutes of relaxation exercises: intersegmental coordination exercises; segmental passive mobilization (until maximum joint range of motion is reached); breathing exercises to promote expansion of the chest.

**Supplementary Material 2 – Kinematic analysis of trunk movement**

Kinematic analysis of trunk was performed with a 4-camera optoelectronic system (SMART DX 400, BTS Engineering, Milan, Italy) with a sampling rate of 100 Hz. Ten spherical reflective markers (15 mm in diameter) were applied at the following sites:

- right acromial process;

- left acromial process;

- spinous process of the 7th cervical vertebra (C7);

- spinous process of the 4th thoracic vertebra;

- spinous process of the 9th thoracic vertebra;

- spinous process of the 12th thoracic vertebra;

- spinous process of the 3rd lumbar vertebra;

- sacral prominence (Sa);

- right anterior-superior iliac spines (ASISr);

- left anterior-superior iliac spines (ASISl).

We studied the patients in static and dynamic conditions. Upright standing position was recorded with subjects standing with their feet 10 cm apart and their arms lying along their trunk. For the dynamic tasks, patients were asked to perform a lateral trunk bending (ipsilateral and contralateral to the side of trunk deviation), a forward trunk flexion and a posterior trunk extension. For each dynamic task they had to reach the maximal range of motion, starting from the upright standing position, and back. Each movement was repeated 4 times, and subjects were allowed to rest as needed between series. The average value of the 4 recordings was used for the analysis.

Synchronized acquisition and data processing were performed using the “SMART analyzer” software (BTS, Milan, Italy). As measurement of lateral and anterior trunk deviation, we considered the absolute deviation of the “C7-Sa” segment from the vector perpendicular to the floor of our movement analysis laboratory during static upright standing position. For the dynamic tasks, we calculated the range of motion (ROM) of trunk, defined as the maximum angle described by the C7-Sa segment starting from upright standing position to the end of each dynamic task.

C7-Sa deviation and ROMs were computed according to a previously validated two-landmark system [7,8]:

1) origin in Sa, X axis is the straight line passing in Sa and the middle point between ASISr and ASISl; Z axis is parallel to the bispinous-iliacae; Y axis is perpendicular to X and Z;

2) origin in Sa, Y axis tending from Sa to C7; X axis perpendicular to the plane made by Sa, C7 and T9; Z axis perpendicular to X and Y.

**Supplementary Material 3 – Results of exploratory endpoints**

- **Exploratory endpoints of kinematic analysis of movement at baseline**

Stat Flex was comparable between t-DCS and sham groups at baseline (p=0.964) (Supplementary Table 1).

ROM Ips (p=0.189), ROM Flex (p=0.554), and ROM Ext (p=0.754) were comparable between t-DCS and sham groups as well. In contrast, the ROM Con was lower in t-DCS group when compared to sham group (p=0.032) (Supplementary Table 1).

- **Effects of t-DCS and sham treatments on exploratory endpoints of the kinematic analysis of trunk parameters**

*Stat Flex* improved significantly after rehabilitation in the overall study population (TIME: p=0.003), without differences between t-DCS and sham groups (STIM: p=0.593). When compared to T0, the *Stat Flex* improvement was significant at T1 (p=0.001 vs. T0), but not at T2 (p=0.223 vs. T0) in both groups (TIMExSTIM: p=0.326) (Supplementary Table 2).

The *ROM Ips* increased after the rehabilitation in the overall population (TIME: p=0.001). The improvement was higher in the t-DCS group (STIM: p=0.037). When compared to T0, the *ROM Ips* improvement was significant at T1 (p=0.001 vs. T0), but not retained at T2 (p=0.079) in both groups (TIMExSTIM: p=0.089) (Supplementary Table 2).

The *ROM Con* increased after the rehabilitation in the overall population (TIME: p=0.027), without significant differences between groups (STIM: p=0.317). When compared to T0, the *ROM Con* improvement was significant at T1 (p=0.022 vs. T0), but not retained at T2 (p=0.791) in both groups (TIMExSTIM: p=0.612) (Supplementary Table 2).

Regarding *ROM Flex*, in t-DCS group we recorded an increase at T1 (p=0.029 vs. T0) which did not persist at T2 (p=1.000 vs. T0). In contrast, we did not detect significant changes in *ROM Flex* in the sham group (p=0.350) (Supplementary Table 2).

The *ROM Ext* was not modified at follow-up evaluations (TIME: p=0.259), and we did not find differences between groups (STIM: p=0.827, and TIMExSTIM: p=0.898) (Supplementary Table 2).

- **Effects of clinical and demographic variables on primary and secondary outcomes**

The kinematic analysis of trunk parameters as well as the scores of the administered questionnaires were not influenced by gender, type of PD at onset, most affected side of PD at onset, or side of trunk deviation.

*Supplementary Table 1 – Baseline parameters of the kinematic analysis of trunk movement: exploratory outcomes*

|  | All patients | t-DCS group | sham group | p-value |
| --- | --- | --- | --- | --- |
| *n* | 28 | 13 | 15 | - |
| Static upright standing position (degree) | | | | |
| Stat Flex | 25.9±13.3 | 25.8±13.5 | 26.1±13.5 | 0.964 |
| Range of motion (ROM) of active dynamic tasks (degree) | | | | |
| ROM Ips | 19.2±10.1 | 16.5±5.9 | 21.5±12.3 | 0.189 |
| ROM Con | 18.4±6.6 | 15.5±6.1 | 20.8±6.3 | 0.032 |
| ROM Flex | 59.4±21.3 | 56.8±23.3 | 61.7±20.0 | 0.554 |
| ROM Ext | 14.3±6.2 | 13.9±6.4 | 14.7±6.1 | 0.754 |

*Legend:* t-DCS: patients randomized to transcranial direct current stimulation (*n*=13). Sham: patients randomized to sham stimulation (*n*=15). Stat Flex: Anterior trunk flexion in the upright standing position. ROM: range of motion. ROM Ips: ROM of trunk bending ipsilateral to the side of trunk deviation. ROM Con: ROM of trunk bending contralateral to the side of trunk deviation. ROM Flex: ROM of anterior trunk flexion. ROM Ext: ROM of posterior trunk extension.

*Supplementary Table 2 –* *Effects of t-DCS and sham treatments on exploratory endpoints of the kinematic analysis of trunk parameters*

|  | T0 | T1 | | | T2 | | | Mixed- model ANOVA | | |
| --- | --- | --- | --- | --- | --- | --- | --- | --- | --- | --- |
|  |  | All patients | t-DCS | sham | All patients | t-DCS | sham | TIME | STIM | TIMExSTIM |
| *n* | 28 | 28 | 13 | 15 | 28 | 13 | 15 |  |  |  |
| Static upright standing position | | | | | | | | | | |
| Stat Flex (%) | 100 | 80.5±13.7 | 72.1±10.8 | 87.8±15.2 | 102.9±27.2 | 104.7±27.2 | 101.5±18.2 | 0.003 | 0.593 | 0.326 |
| Range of motion (ROM) of active dynamic tasks | | | | | | | | | | |
| ROM Ips (%) | 100 | 132.3±17.1 | 147.0±17.0 | 119.6±14.9 | 117.2±20.7 | 129.8±23.6 | 106.2±16.7 | 0.001 | 0.037 | 0.089 |
| ROM Con (%) | 100 | 125.2±23.2 | 133.0±22.9 | 118.4±23.7 | 111.5±28.2 | 122.3±38.3 | 102.1±14.9 | 0.027 | 0.317 | 0.612 |
| ROM Flex (%) | 100 | 107.8±26.7 | 131.3±27.8 | 87.4±21.7 | 102.9±27.2 | 104.7±35.8 | 101.5±18.2 | 0,520 | 0.214 | 0.014 |
| ROM Ext (%) | 100 | 127.2±40.5 | 120.4±34.7 | 133.1±45.9 | 111.5±33.4 | 114.9±44.0 | 108.5±22.1 | 0.259 | 0.827 | 0.898 |

*Legend:* t-DCS: patients randomized to transcranial direct current stimulation (*n*=13). Sham: patients randomized to sham stimulation (*n*=15). Stat Flex: Anterior trunk flexion in the upright standing position. ROM: range of motion. ROM Ips: ROM of trunk bending ipsilateral to the side of trunk deviation. ROM Con: ROM of trunk bending contralateral to the side of trunk deviation. ROM Flex: ROM of anterior trunk flexion. ROM Ext: ROM of posterior trunk extension. Mixed-model *ANOVA:* factor “TIME” is expression of the efficacy of the rehabilitative treatment in the overall population; factor “STIM” is expression of the comparison between t-DCS and sham groups across all time-points; a significant TIMExSTIM interaction is expression of a difference between t-DCS and sham groups as well as difference in the persistence of the effects between t-DCS and sham groups over time.

*Supplementary Table 3 – Distribution of Item 3.13 Posture of the UPDRS-III in t-DCS and sham groups*

|  | T0 | | T1 | | T2 | | Pearson χ^2^ test (t-DCS vs. sham) | | |
| --- | --- | --- | --- | --- | --- | --- | --- | --- | --- |
|  | t-DCS | sham | t-DCS | sham | t-DCS | sham | T0 | T1 | T2 |
| n | 13 | 15 | 13 | 15 | 13 | 15 |  |  |  |
| Score 1 | 1 (7.7%) | 1 (6.7%) | 8 (61.5%) | 1 (6.7%) | 4 (30.8%) | 1 (6.7%) | 0.892 | 0.019 | 0.355 |
| Score 2 | 5 (38.5%) | 6 (40.0%) | 4 (30.8%) | 10 (66.7%) | 6 (42.6%) | 8 (53.3%) |  |  |  |
| Score 3 | 5 (38.5%) | 7 (46.7%) | 1 (7.7%) | 3 (20.0%) | 2 (15.4%) | 5 (33.3%) |  |  |  |
| Score 4 | 2 (15.4%) | 1 (6.7%) | 0 (0.0%) | 1 (6.7%) | 1 (7.7%) | 1 (6.7%) |  |  |  |

Legend: t-DCS: patients randomized to transcranial direct current stimulation (n=13). Sham: patients randomized to sham stimulation (n=15). UPDRS-III: Unified Parkinson’s Disease Rating Scale – part III – Motor examination.

**Bibliography**

[1] A.F. Dasilva, M.S. Volz, M. Bikson, F. Fregni, Electrode Positioning and Montage in Transcranial Direct Current Stimulation, J. Vis. Exp. (2011) 2744. https://doi.org/10.3791/2744.

[2] J. Rosset-Llobet, S. Fàbregas-Molas, Á. Pascual-Leone, Effect of Transcranial Direct Current Stimulation on Neurorehabilitation of Task-Specific Dystonia: A Double-Blind, Randomized Clinical Trial, Med. Probl. Perform. Art. 30 (2015) 178–184. https://doi.org/10.21091/mppa.2015.3033.

[3] C. Swank, J. Mehta, C. Criminger, Transcranial direct current stimulation lessens dual task cost in people with Parkinson’s disease, Neurosci. Lett. 626 (2016) 1–5. https://doi.org/10.1016/j.neulet.2016.05.010.

[4] A.J. Woods, A. Antal, M. Bikson, P.S. Boggio, A.R. Brunoni, P. Celnik, L.G. Cohen, F. Fregni, C.S. Herrmann, E.S. Kappenman, H. Knotkova, D. Liebetanz, C. Miniussi, P.C. Miranda, W. Paulus, A. Priori, D. Reato, C. Stagg, N. Wenderoth, M.A. Nitsche, A.J. Woods, C.N. Author, A technical guide to tDCS, and related non-invasive brain stimulation tools HHS Public Access Author manuscript, Clin Neurophysiol. 127 (2016) 1031–1048. https://doi.org/10.1016/j.clinph.2015.11.012.

[5] G. Castelnuovo, E.M. Giusti, G.M. Manzoni, D. Saviola, S. Gabrielli, M. Lacerenza, G. Pietrabissa, R. Cattivelli, C.A. Maria Spatola, A. Rossi, G. Varallo, M. Novelli, V. Villa, F. Luzzati, A. Cottini, C. Lai, E. Volpato, C. Cavalera, F. Pagnini, V. Tesio, L. Castelli, M. Tavola, R. Torta, M. Arreghini, L. Zanini, A. Brunani, I. Seitanidis, G. Ventura, P. Capodaglio, G.E. D’Aniello, F. Scarpina, A. Brioschi, M. Bigoni, L. Priano, A. Mauro, G. Riva, D. Di Lernia, C. Repetto, C. Regalia, E. Molinari, P. Notaro, S. Paolucci, G. Sandrini, S. Simpson, B.K. Wiederhold, S. Gaudio, J.B. Jackson, S. Tamburin, F. Benedetti, M. Agostini, E. Alfonsi, A.M. Aloisi, E. Alvisi, I. Aprile, M. Armando, M. Avenali, E. Azicnuda, F. Barale, M. Bartolo, R. Bergamaschi, M. Berlangieri, V. Berlincioni, L. Berliocchi, E. Berra, G. Berto, S. Bonadiman, S. Bonazza, F. Bressi, A. Brugnera, S. Brunelli, M.G. Buzzi, C. Cacciatori, A. Calvo, C. Cantarella, A. Caraceni, R. Carone, E. Carraro, R. Casale, P. Castellazzi, A. Castino, R. Cerbo, A. Chiò, C. Ciotti, C. Cisari, D. Coraci, E. Dalla Toffola, G. Defazio, R. De Icco, U. Del Carro, A. Dell’Isola, A. De Tanti, M. D’Ippolito, E. Fazzi, A. Ferrari, S. Ferrari, F. Ferraro, F. Formaglio, R. Formisano, S. Franzoni, F. Gajofatto, M. Gandolfi, B. Gardella, P. Geppetti, A. Giammò, R. Gimigliano, E. Greco, V. Ieraci, M. Invernizzi, M. Jacopetti, S. La Cesa, D. Lobba, F. Magrinelli, S. Mandrini, U. Manera, P. Marchettini, E. Marchioni, S. Mariotto, A. Martinuzzi, M. Masciullo, S. Mezzarobba, D. Miotti, A. Modenese, M. Molinari, S. Monaco, G. Morone, R. Nappi, S. Negrini, A. Pace, L. Padua, E. Pagliano, V. Palmerini, C. Pazzaglia, C. Pecchioli, A. Picelli, C.A. Porro, D. Porru, M. Romano, L. Roncari, R. Rosa, M. Saccavini, P. Sacerdote, A. Schenone, V. Schweiger, G. Scivoletto, N. Smania, C. Solaro, V. Spallone, I. Springhetti, C. Tassorelli, M. Tinazzi, R. Togni, M. Torre, M. Traballesi, M. Tramontano, A. Truini, V. Tugnoli, A. Turolla, G. Vallies, E. Verzini, M. Vottero, P. Zerbinati, What is the role of the placebo effect for pain relief in neurorehabilitation? Clinical implications from the Italian consensus conference on pain in neurorehabilitation, Front. Neurol. 9 (2018). https://doi.org/10.3389/fneur.2018.00310.

[6] G.N. Lewis, D.A. Rice, M. Kluger, P.J. McNair, Transcranial direct current stimulation for upper limb neuropathic pain: A double-blind randomized controlled trial, Eur. J. Pain (United Kingdom). 22 (2018) 1312–1320. https://doi.org/10.1002/ejp.1220.

[7] M. Bartolo, M. Serrao, C. Tassorelli, R. Don, A. Ranavolo, F. Draicchio, C. Pacchetti, S. Buscone, A. Perrotta, A. Furnari, P. Bramanti, L. Padua, F. Pierelli, G. Sandrini, Four-week trunk-specific rehabilitation treatment improves lateral trunk flexion in Parkinson’s disease, Mov. Disord. 25 (2010) 325–331. https://doi.org/10.1002/mds.23007.

[8] C. Tassorelli, R. De Icco, E. Alfonsi, M. Bartolo, M. Serrao, M. Avenali, I. De Paoli, C. Conte, N.G. Pozzi, P. Bramanti, G. Nappi, G. Sandrini, Botulinum toxin type A potentiates the effect of neuromotor rehabilitation of Pisa syndrome in Parkinson disease: A placebo controlled study, Park. Relat. Disord. 20 (2014) 1140–1144. https://doi.org/10.1016/j.parkreldis.2014.07.015.
